# PD-L1 induction via the MEK-JNK-AP1 axis by a neddylation inhibitor promotes cancer-associated immunosuppression

**DOI:** 10.1038/s41419-022-05292-9

**Published:** 2022-10-03

**Authors:** Shizhen Zhang, Xiahong You, Tiantian Xu, Qian Chen, Hua Li, Longyu Dou, Yilun Sun, Xiufang Xiong, Morgan A. Meredith, Yi Sun

**Affiliations:** 1 Cancer Institute, the Second Affiliated Hospital, Zhejiang University School of Medicine, 310029 Hangzhou, China; 2 Department of Breast Surgery and Oncology, Key Laboratory of Cancer Prevention and Intervention, Ministry of Education, the Second Affiliated Hospital, Zhejiang University School of Medicine, 310029 Hangzhou, China; 3 Institute of Translational Medicine, Zhejiang University School of Medicine, 310029 Hangzhou, China; 4 Division of Radiation and Cancer Biology, Department of Radiation Oncology, University of Michigan, 4424B MS-1, 1301 Catherine Street, Ann Arbor, MI 48109 USA; 5 Zhejiang University Cancer Center, 310029 Hangzhou, China; 6 Research Center for Life Science and Human Health, Binjiang Institute of Zhejiang University, Hangzhou, 310053 Zhejiang China

**Keywords:** Tumour immunology, Cell signalling

## Abstract

MLN4924 is a first-in-class small molecule inhibitor of NEDD8-activating enzyme (NAE), which is currently in several clinical trials for anti-cancer applications. However, MLN4924 also showed some off-target effects with potential to promote the growth of cancer cells which counteracts its anticancer activity. In this study, we found that MLN4924 increases the levels of PD-L1 mRNA and protein in dose- and time-dependent manners. Mechanistic study showed that this MLN4924 effect is largely independent of neddylation inactivation, but is due to activation of both ERK and JNK signals, leading to AP-1 activation, which is blocked by the small molecule inhibitors of MEK and JNK, respectively. Biologically, MLN4924 attenuates T cell killing in a co-culture model due to PD-L1 upregulation, which can be, at least in part, abrogated by either MEK inhibitor or anti-PD-L1 antibody. In an in vivo BALB/c mouse xenograft tumor model, while MLN4924 alone had no effect, combination with either MEK inhibitor or anti-PD-L1 antibody enhanced the suppression of tumor growth. Taken together, our study provides a sound rationale for effective anticancer therapy in combination of anti-PD-L1 antibody or MEK inhibitor with MLN4924 to overcome the side-effect of immunosuppression by MLN4924 *via* PD-L1 induction.

## Introduction

Protein neddylation is an important biochemical process by which the ubiquitin-like protein NEDD8 (Neural precursor cell-expressed developmentally down-regulated gene 8) is covalently attached to a substrate protein to affect its function, not degradation. Like ubiquitylation, neddylation is catalyzed by three-step enzymatic cascade of E1 NEDD8-activating enzyme (NAE), NEDD8-conjuagating enzyme E2 and substrate-specific NEDD8-E3 ligases [1–3]. The physiological substrates of neddylation are the cullin family members and cullin neddylation is required for the activity of cullin-RING ligases (CRLs); CRLs control the ubiquitylation of ~20% proteins doomed to be degraded *via* proteasome, thus regulating many important biological processes [4]. In the past decades, numerous studies have convincingly demonstrated that the neddylation pathway and CRLs are significantly over-activated in various types of human cancers, and have been validated as attractive anti-cancer targets [5].

MLN4924 (also known as pevonedistat) is a first-in-class NAE inhibitor reported in 2009 [6] with a mechanism involving the formation of a steady-state covalent adduct between the ATP-binding site of NAE and C-terminal of NEDD8, thus blocking NEDD8 transferring to neddylation E2 [7], leading to inactivation of the entire family of CRLs [6–8]. In many preclinical studies, MLN4924 has demonstrated impressive anticancer activity, both acting alone or in combination with chemotherapy, radiotherapy and immunotherapy [5, 9], which led to a series of phase I/II/III clinical trials in the treatment of patients with both hematologic and solid malignancies, alone or in combination with various chemotherapeutic agents [10–13]. However, recent preclinical studies have shown that MLN4924 suffered from a number of off-target effects, which compromises its anticancer activity [14].

T cells play an important role in the maintenance of the immune system and in the anti-tumor immune response. Co-inhibitory immune checkpoint proteins, such as cytotoxic T-lymphocyte-associated protein 4 (CTLA-4), programmed death-1 (PD-1), and programmed death-ligand (PD-L1) are critical for maintaining a balanced regulation of CD8^+^ T-cell activities, since uncontrolled hyper-activation of these T cell population may result in autoimmune reactions [15]. It is well-established that tumor-associated immunosuppression is a major barrier for the success of immunotherapy [16]. A critical mechanism underlying cancer-associated immunosuppression is the aberrant expression of PD-L1 on the surface of cancer cells. Engagement between PD-1 receptor expressed on T cells and PD-L1 on tumor cells contributes to the suppression of T cell activity and cytokine release, eventually leading to immunosuppression [17]. Blockade of PD-1 or PD-L1, therefore, is able to restore T cell functions and potentiate therapeutic efficacy [18]. Multiple conventional and targeted chemotherapies could modulate anticancer immunity and affect tumor-targeting immune responses. For example, chemotherapy regimens, FLOT (5-FU, oxaliplatin, docetaxel) and CROSS (carboplatin and paclitaxel) could promote an immune-resistant phenotype through upregulation of inhibitory immune checkpoint ligands and receptors, such as PD-1/PD-L1 [19]; PARP inhibitor could enhance cancer-associated immunosuppression by upregulating PD-L1 expression [20]; MAP kinase inhibitor is able to potentiate antitumor T cells by blocking TCR-driven apoptosis [21]. Thus, the combination of cytotoxic or targeted anticancer therapies with immune checkpoint blockade treatment would result in more promising and potent anticancer effect.

Two recent studies showed that PD-L1 is induced by MLN4924 treatment in glioma cells [9, 22], suggesting that the levels of PD-L1 are subjected to regulation by neddylation. PD-L1 was also found to be a substrate of CRL3^SPOP^ E3 ligase upon phosphorylation by cyclin D-CDK4 [23], whereas MLN4924 increased PD-L1 levels by inactivating CRL1^FBXW7^, leading to accumulation of c-MYC, which trans-activated PD-L1 expression [9]. In this study, we reported that in multiple human cancer cell lines, MLN4924 increased PD-L1 expression in dose- and time-dependent manners mainly through the activation of transcription factor AP-1 *via* MEK signals, leading to the establishment of a cancer-associated immunosuppression environment. The blockade of PD-L1 *via* anti-PD-L1 antibody or MEK inhibitor restored the attenuated antitumor immunity, resulting in an enhanced tumor suppression both in vitro co-culture and in vivo animal models. Our study revealed a neddylation-independent side-effect of the neddylation inhibitor MLN4924, and provided a sound strategy for effective combinational anti-cancer therapy.

## Results

### MLN4924 enhances PD-L1 expression at both mRNA and protein levels in dose- and time-dependent manners

**Fig. 1:**
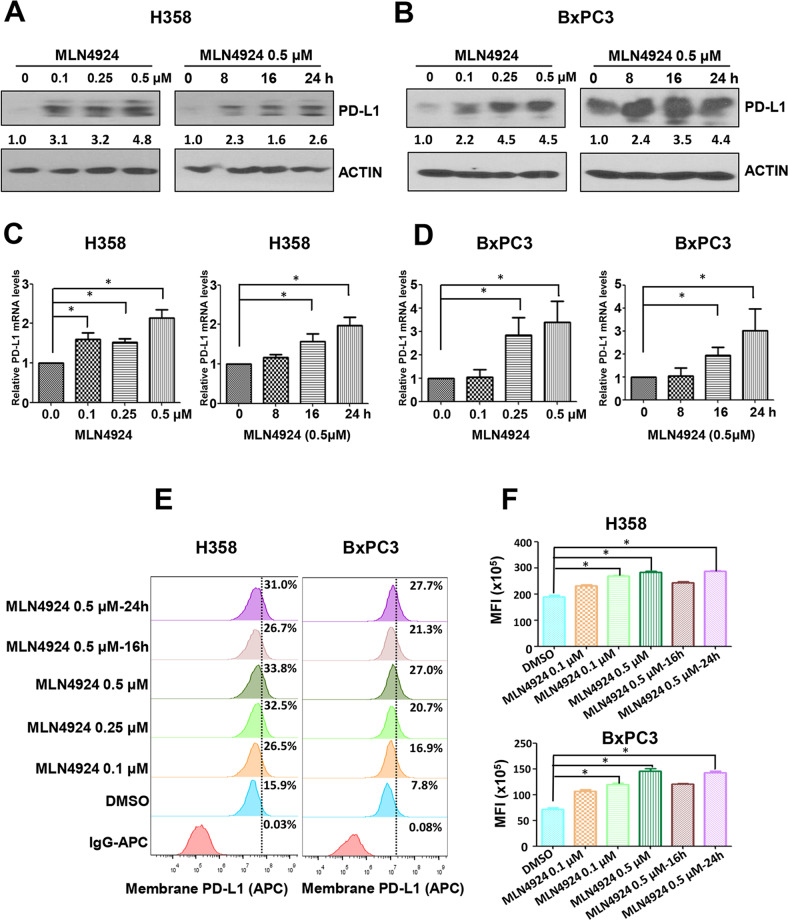
MLN4924 increases PD-L1 expression at both mRNA and protein levels in dose- and time-dependent manners. **A–D** MLN4924 treatment increases PD-L1 protein levels and mRNA levels in dose- and time-dependent manners. H358 and BxPC3 Cells were treated with various concentrations of MLN4924 for the indicated time periods, followed by IB with the indicated Abs (**A**, **B**), or followed by qRT-PCR analysis (**C**, **D**). **E**, **F** MLN4924 treatment increases the cell surface PD-L1 levels in dose- and time-dependent manners. H358 and BxPC3 Cells were treated with various concentrations of MLN4924 for the indicated time periods, followed by FACS analysis. MFI Median Fluorescence Intensity; *p* < 0.05 (*).

Two previous studies showed that MLN4924 induced PD-L1 expression in glioblastoma cells [9, 22]. To determine whether this is a general phenomenon, we extended the observation and found that MLN4924 increased the levels of PD-L1 protein in multiple human cancer cell lines, including esophageal and head & neck cancer cells (Kyse70, UMSCC1, UMSCC38, UMSCC5, UMSCC10, UMSCC11b, UMSCC17); pancreatic cancer cells (Moh1, MIAPaCa2, BxPC3) and lung cancer cells (H358, H1792, H460, A549, H1650, H1299) (Fig. S1A–E), as well as few murine cancer cell lines, B16-F10, MC38 and CT26 (Fig. S1F), indicating a general phenomenon. We then focused on H358 lung cancer cells and BxPC3 pancreatic cancer cells with PD-L1 at relatively low basal levels and inducible by MLN4924, and found MLN4924 increased PD-L1 expression at both protein and mRNA levels in dose- and time-dependent manners (Fig. 1A–D). A time-dependent induction of PD-L1 protein levels by MLN4924 was also seen in SK-MES-1 lung cancer cells with low basal level of PD-L1 (Fig. S1G). Given that the cell surface PD-L1 expressed on cancer cells mainly dominates the anticancer immunosuppression [24], we determined whether the cell surface PD-L1 was increased after MLN4924 treatment, using the fluorescence-labeled anti-PD-L1 antibody for FACS analysis. Significantly, cell surface PD-L1 levels were indeed increased upon MLN4924 treatment in dose- and time-dependent manners (Fig. 1E, F). Collectively, these results showed that MLN4924, when applied for cancer treatment, would upregulate PD-L1 expression on cancer cell surface with potential to trigger anticancer immunosuppression, as a major side-effect.

### PD-L1 induction by MLN4924 is largely neddylation-independent, but the ERK1/2-JNK pathway-dependent

**Fig. 2:**
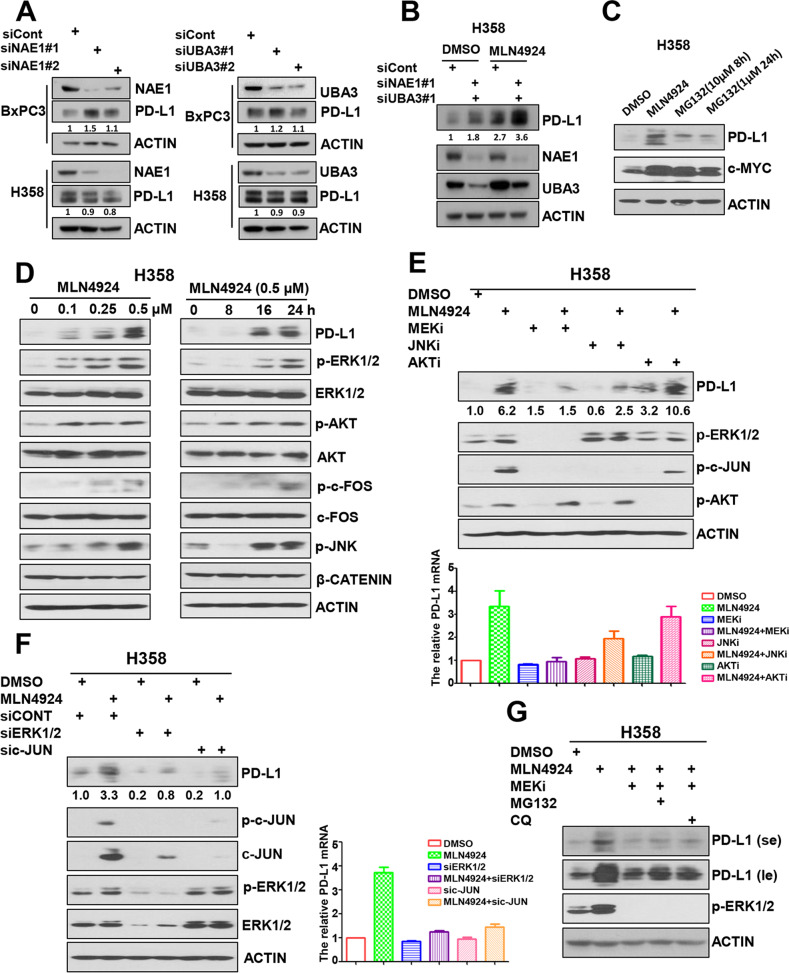
MLN4924 induction of PD-L1 is neddylation-independent, but ERK1/2-JNK pathway-dependent. **A** Knockdown of *NAE1* or *UBA3* had a minor effect on PD-L1 levels. H358 and BxPC3 cells were transfected with siRNAs targeting NAE1, UBA3 or control siCont for 48 h, followed by IB with the indicated Abs. **B** *NAE1* or *UBA3* knockdown had a minor effect on MLN4924-induced PD-L1 induction. H358 cells were transfected with indicated siRNAs for 24 h, then cells were treated with the indicated concentrations of MLN4924 for 24 h, followed by IB with the indicated Abs. **C** Proteasome inhibitor MG132 had minor effect on PD-L1 levels. H358 cells were treated with MLN4924 (0.5 μM) for 24 h or MG132 (10 μM) for 8 h or MG132(1 μM) for 24 h, followed by IB with the indicated Abs. **D** MLN4924 induced the phosphorylation of ERK1/2, AKT, JNK and c-FOS in dose- and time-dependent manners. H358 cells were treated with various concentrations of MLN4924 for the indicated time periods, followed by IB with the indicated Abs. **E** MLN4924-induced elevation of both PD-L1 protein (Top) and mRNA (Bottom) can be largely abrogated by MEK inhibitor (trametinib) or JNK inhibitor (SP600125). H358 cells were treated with either MLN4924 or indicated inhibitors or both, followed by IB with the indicated Abs. **F** MLN4924 induction of both PD-L1 protein (Left) and mRNA (Right) can be largely abrogated by knockdown of *ERK1/*2, or *c-JUN*. H358 cells were transfected with siRNAs targeting ERK1/2, c-JUN or control siRNA, followed by treatment with MLN4924 (0.5 μM) for 24 h, then for IB with the indicated Abs. **G** MEK inhibitor-induced abrogation of PD-L1 induction occurred at the transcriptional level. H358 cells were treated with MLN4924 alone or combined with indicated inhibitors, followed by IB with the indicated Abs. MEKi MEK inhibitor (trametinib), JNKi JNK inhibitor (SP600125), AKTi AKT inhibitor (MK2206), MG132 proteasome inhibitor, CQ (chloroquine) lysosome inhibitor, se short exposure, le long exposure.

Given that MLN4924 is a specific NAE inhibitor [6, 7], we determined if siRNA-based knockdown of NAE would achieve the same PD-L1 increase. Surprisingly and unexpectedly, knockdown of either *NAE1* or *UBA3* or both had a minor effect on PD-L1 levels and minor effect on MLN4924-induced PD-L1 increase (Figs. 2A, B and S2A, B). Moreover, unlike MLN4924, the proteasome inhibitor MG132 had minor effect on PD-L1 levels, while having equal capacity in causing accumulation of c-MYC, a classical substrate of CRL1, followed by proteasome degradation (Figs. 2C and S2C), suggesting (1) that PD-L1 accumulation by MLN4924 was not completely attributable to the blockage of neddylation-CRL pathway, (2) the possible involvement of another neddylation-independent mechanism.

Our previous study showed that MLN4924 had an off-target effect of inducing EGFR dimerization to activate EGFR and its downstream pathways, including the PI3K/AKT and MAPK signaling pathways [14, 25]. Indeed, in both H358 lung cancer and BxPC3 pancreatic cancer cells, MLN4924 induced PD-L1, and at the same time the phosphorylation of ERK1/2, AKT and JNK kinases as well as c-FOS in dose- and time- dependent manners (Figs. 2D and S2D), indicating the activation of these signaling pathways. Consistently, MLN4924-induced elevation of both PD-L1 protein and mRNA was largely abrogated by MEK inhibitor (trametinib), JNK inhibitor (SP600125), as well as knockdown of *ERK1/2*, or *c-JUN*, but not by AKT inhibitor (MK2206) (Figs. 2E, F and S2E, F), indicating that the activation of ERK1/2-JNK pathways, but not the AKT pathway mediates the induction of PD-L1 by MLN4924. Interestingly, MEK inhibitor-induced abrogation of PD-L1 induction could not be blocked by either proteasome inhibitor MG132 or the lysosome inhibitor CQ (Figs. 2G and S2G), indicating that MAPK pathway upregulation of PD-L1 occurs at the transcriptional level and not through the proteasomal or lysosomal degradation pathways. In order to determine whether MLN4924 induction of PD-L1 through ERK1/2-JNK pathway is in a dose-dependent manner, we first generated MLN4924 IC_50_ curve in both H358 and BxPC3 cells (Fig. S3A, B), and then measured PD-L1 induction by MLN4924 at the broad range of concentrations (IC_20/40/60/80_) in the absence or presence of MEK inhibitor/JNK inhibitor. The results showed that inhibitors of MEK or JNK abrogated MLN4924 induction of PD-L1 across all tested concentrations (Fig. S3C–F). Our results showed that the ERK1/2-JNK axis plays the major role in MLN4924 induction of PD-L1 in a manner largely independent of MLN4924 concentrations in both H358 and BxPC3 cells, whereas the neddylation-CRL ubiquitylation-degradation pathway [9, 23] plays a minor role under our experimental conditions.

### AP-1 activity controls the MLN4924 induction of PD-L1

**Fig. 3:**
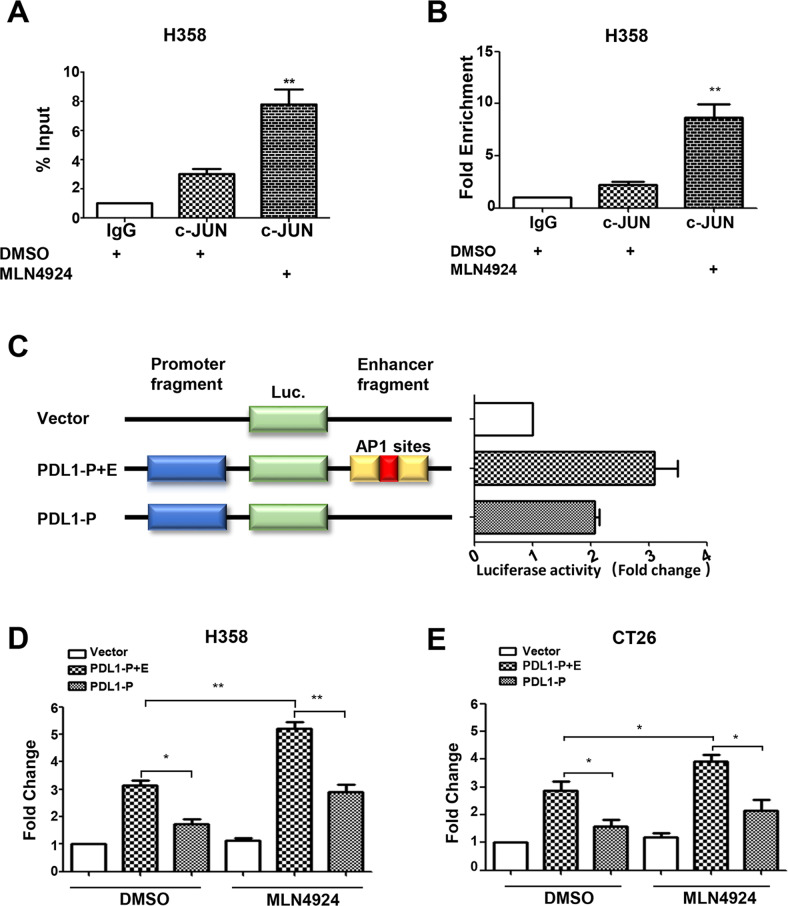
AP-1 activity controls the MLN4924 induction of PD-L1 expression. **A**, **B** c-JUN bound to the PD-L1 enhancer sequence and the binding was significantly enhanced by MLN4924 treatment. H358 cells were treated with MLN4924 (0.5 μM) for 24 h, followed by ChIP-coupled PCR assay. The results were represented as % input (**A**) and Fold Enrichment (**B**). **C** AP-1-containing enhancer element increased PD-L1 promoter-driven luciferase activity. Constructs include the empty pGL3 vector, the pGL3 vector with the promoter cloned upstream of the luciferase gene alone (PDL1-P), the promoter with the wild-type enhancer cloned downstream of the luciferase gene (PDL1-P + E). **D**, **E** MLN4924 induction of PD-L1 depends on AP-1 binding site. H358 (**D**) and CT26 (**E**) cells were transfected with the indicated plasmids for 24 h, and then treated with MLN4924 (0.5 μM) for 24 h, followed by luciferase-based transcriptional assay. Shown are mean ± SEM of three independent experiments. *p* < 0.05 (*), *p* < 0.01 (**).

The MAPK-regulated c-JUN/c-FOS heterodimer is the prototypic activator protein 1 (AP-1) member [26], which binds to the AP-1 binding site to transactivate target gene expression [27]. We next focused on potential transcriptional regulation of PD-L1 by AP-1. Indeed, the tandem AP-1 binding sites were reported at the highly conserved enhancer element of the *PD-L1* gene [28, 29]. In a ChIP-coupled PCR assay, we found that c-JUN indeed bound to the *PD-L1* enhancer sequence and the binding was significantly enhanced by MLN4924 treatment in both H358 and BxPC3 cells (Figs. 3A, B and S4A, B). To assess the activity of the AP-1 enhancer element, we conducted the luciferase-based transcriptional assay, using the pGL3 luciferase plasmids containing either the *PD-L1* promoter element (PDL1-P) or the promoter element plus the AP-1-containing enhancer element (PDL1-P + E). The results showed that the AP-1-containing enhancer element increased *PD-L1* promoter-driven luciferase activity in an AP-1 dependent manner (Fig. 3C), and such luciferase activity was significantly increased after MLN4924 treatment for 24 h in both human cancer cell lines (H358, BxPC3) (Figs. 3D and S4C) and a murine colon cancer cell line CT26 (Fig. 3E). Moreover, prolonged MLN4924 treatment (48 h) also increased the *PD-L1* promoter-driven luciferase activity in both H358 and BxPC3 cells (Fig. S4D, E). Collectively, MLN4924 increases PD-L1 expression through AP-1 activation and in an AP-1 dependent manner.

### MLN4924 attenuates cytotoxic effect of Jurkat T cells through PD-L1 induction

**Fig. 4:**
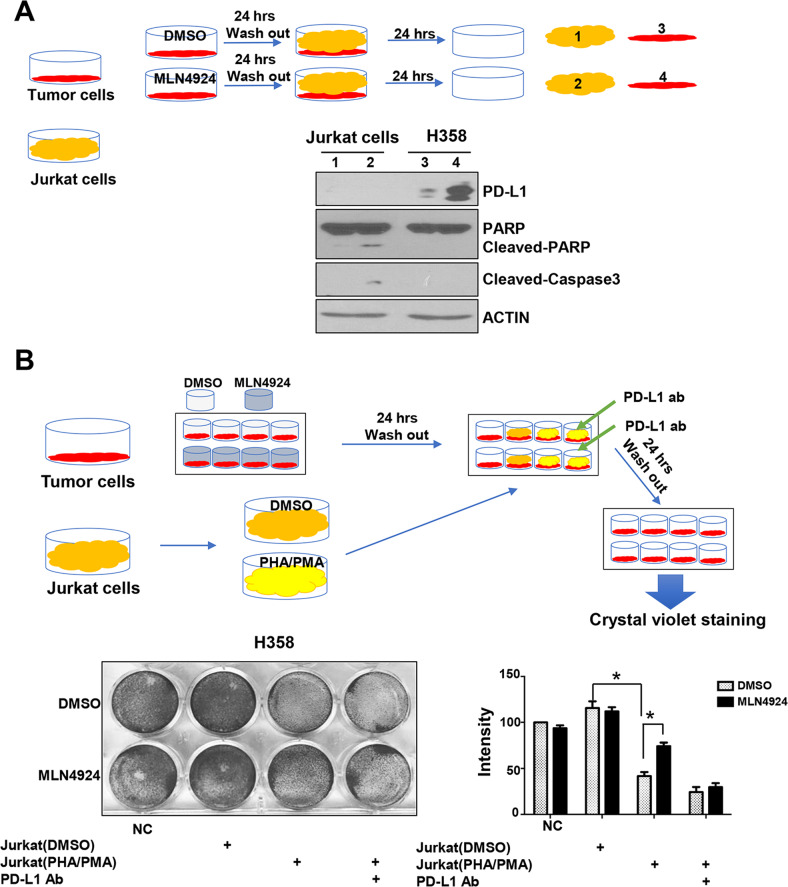
MLN4924 attenuates cytotoxic effect of Jurkat cells through PD-L1 induction. **A** Jurkat cells co-cultured with MLN4924-pretreated cancer cells underwent apoptosis. Sub-confluent H358 cells were treated with MLN4924 (0.5 μM) for 24 h. After medial removal and PBS washing, suspension of Jurkat cells was added and cultured for 24 h. H358 and Jurkat cells were then harvested, separately, for IB with the indicated Abs. **B** MLN4924-pretreated cancer cells became resistant to the killing by activated Jurkat cells. H358 cells were treated with MLN4924 (0.5 μM) for 24 h, co-cultured with PHA/PMA-activated Jurkat cells with or without anti-PD-L1 antibody (PD-L1 ab) for 24 h, followed by crystal violet staining of viable adhered H358 cells and photography. Shown are mean ± SEM of three independent experiments. *p* < 0.05 (*), *p* < 0.01 (**).

To understand the potential biological consequence of PD-L1 upregulation by MLN4924, we asked whether MLN4924 induction of PD-L1 in cancer cells would negatively affect the viability and function of T cells, and performed a co-culture assay of the Jurkat T cells with cancer cells with or without MLN4924 pretreatment (Figs. 4A and S5A, top panel). As expected, cancer cells with MLN4924-pretreatment had a higher PD-L1 expression. Interestingly, Jurkat cells co-cultured with MLN4924-pretreated cancer cells underwent apoptosis, although the effect is minor, as evidenced by minor increased levels of cleaved-PARP and cleaved caspase-3 (Figs. 4A and S5A), suggesting that PD-L1 induction by MLN4924 in cancer cells indeed triggers the killing of co-cultured Jurkat cells.

Next, we determined the killing effect of activated Jurkat cells on cancer cells with or without MLN4924 pretreatment and found that the Jurkat cells activated by co-stimulation of PHA (Phytohaemaglutinin) and PMA (phorbol myristate acetate) [30] indeed effectively killed co-cultured cancer cells, but the effect was much compromised upon MLN4924 pretreatment (Figs. 4B and S5B), indicating that MLN4924-pretreated cancer cells became resistant to the killing induced by activated Jurkat cells. We then determined if this resistance was mediated by PD-L1 upregulation, and found that the killing effect of Jurkat cells against MLN4924-pretreated cancer cells was completely restored upon inclusion of anti-PD-L1 antibody (Figs. 4B and S5B). All these results supported the notion that MLN4924 induction of PD-L1 on the surface of cancer cells, on one hand, triggers T cell death, and on the other hand, inhibits the cytotoxic activity of the T cells against the cancer cells, leading to resistance of cancer cells to T cell killing, which is re-sensitized by anti-PD-L1 antibody, indicating a causal role of PD-L1.

### MEK inhibitor enhances cytotoxic effect of Jurkat cells on MLN4924-pretreated cancer cells

**Fig. 5:**
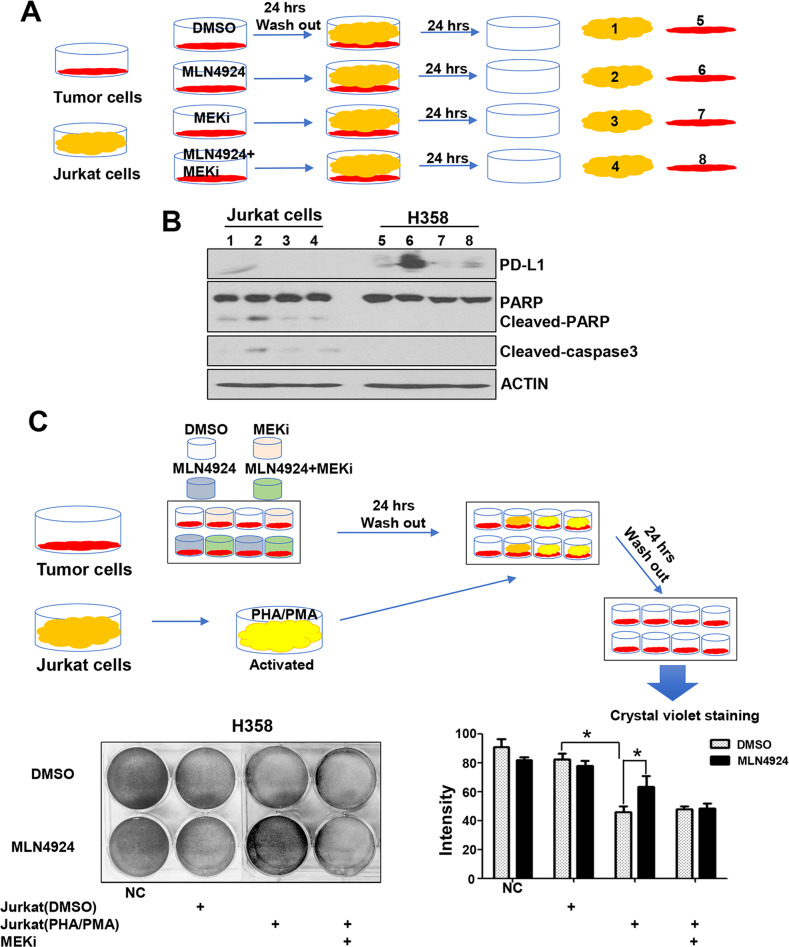
MEK inhibitor enhances cytotoxic effect of Jurkat cells on MLN4924-pretreated cancer cells. **A**, **B** H358 cells were treated with either MLN4924 (0.5 μM) or indicated inhibitors or both for 24 h, then co-cultured with Jurkat cells for 24 h. The H358 and Jurkat cells were separately harvested for IB with the indicated Abs. **C** H358 cells were treated with either MLN4924 (0.5 μM) or indicated inhibitors or both for 24 h, co-cultured with PHA/PMA-activated Jurkat cells for 24 h, followed by crystal violet staining and photography. Shown are mean ± SEM of three independent experiments. *p* < 0.05 (*), *p* < 0.01 (**). NC negative control without Jurkat co-culture nor drug treatment.

Given that MEK inhibitor trametinib abrogated the MLN4924-induced PD-L1 upregulation (Fig. 2E), we next determined whether MEK inhibitor would also re-sensitize MLN4924-pretreated cancer cells to Jurkat cell killing. Indeed, MEK inhibitor significantly inhibited PD-L1 expression in cancer cells pretreated with MLN4924, along with inhibition of apoptosis of co-cultured Jurkat cells (Figs. 5A, B and S6A). Consequently, MEK inhibitor re-sensitized MLN4924-pretreated cancer cells to the killing induced by activated Jurkat cells (Figs. 5C and S6B). It appears that the PD-L1 induction by MLN4924 plays a causal role, at least in part, for MEK inhibitor resensitization of tumor cells to T cell-mediated killing.

### Combination of MLN4924 with MEK inhibitor or anti-PD-L1 antibody enhanced tumor suppression in a synergistic BALB/c xenograft tumor model

**Fig. 6:**
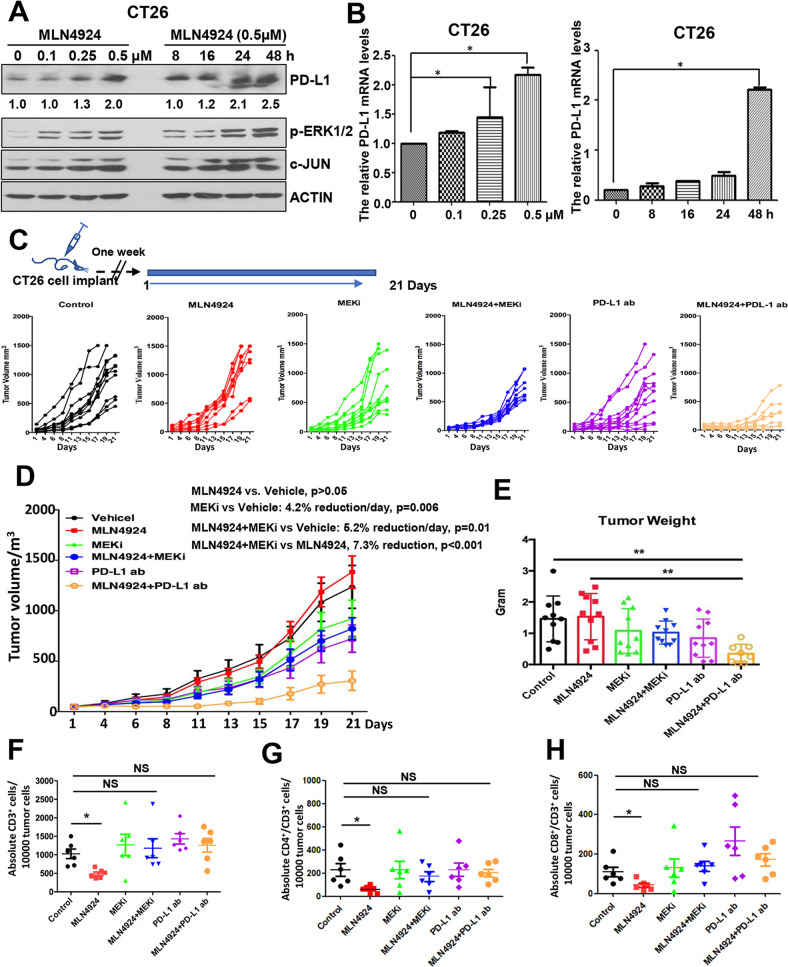
Enhanced tumor suppression by combination MLN4924 with MEK inhibitor or anti-PD-L1 antibody in xenograft tumor model. **A**, **B** MLN4924 treatment increases PD-L1 mRNA and protein levels in dose- and time-dependent manners. CT26 cells were treated with various concentrations of MLN4924 for indicated time periods, followed by IB and qRT-PCR analysis. **C**, **D** Tumor growth rate. Top: drug treatment regimen. Bottom: tumor growth rate after 6 indicated treatments (Control, MLN4924, MEKi, PD-L1 ab, MLN4924 + MEKi, MLN4924 + PD-L1 ab) at each individual (**C**) or group level (**D**). **E** The weight of tumors after harvesting at the end of experiment. **F**–**H** FACS profiling of tumor infiltrated T-cell population: Single cell suspension of tumor mass was prepared from each treatment group, and subjected to FACS profiling with indicated Abs. The data were quantified and statistically analyzed. MEKi MEK inhibitor (trametinib), PD-L1 ab anti-PD-L1 antibody; *p* < 0.05 (*), *p* < 0.01 (**); NS not significant.

It has been reported that chemotherapy-induced PD-L1 upregulation contributes to drug resistance due to PD-L1 induced immune-suppression [31]. Given MLN4924 upregulated PD-L1 expression through activation of MEK, we hypothesized that this MLN4924 side-effect would compromise its anti-cancer activity, and by blocking PD-L1, MEK inhibitor or anti-PD-L1 antibody should overcome this side-effect. To test this hypothesis in vivo, we generated a xenograft CT26 mouse colon cancer model in immune-competent synergistic BALB/c mice. We first confirmed that in CT26 cells, MLN4924 indeed induced PD-L1 at both mRNA and protein levels as well as ERK1/2 activation, which can be blocked by MEK/JNK inhibitors (Figs. 6A, B and S7A). While MLN4924 hardly inhibited CT26 tumor growth, and either MEK inhibitor or anti-PD-L1 antibody moderately restricted tumor growth, the combination of MLN4924 with MEK inhibitor or particularly with anti-PD-L1 antibody significantly enhanced suppression of tumor growth (Figs. 6C–E and S7B).

We next determined tumor infiltration of immune cells in each treatment group. Tumor tissues were resected, lysed for Western blotting or single cell suspension prepared for FACS profiling. Western blot analysis of the tumor lysis showed that MLN4924-treated tumors had in general upregulated PD-L1 expression, which was lower in MEK inhibitor-treated tumors (Fig. S7C). The FACS analysis of tumor mass revealed that while tumor-infiltrating T-cell populations, including CD3^+^, CD3^+^/CD4^+^, CD3^+^/CD8^+^ cells were significantly lower upon MLN4924 treatment, the combination with MEK inhibitor or anti-PD-L1 antibody restored the cytotoxic T-cell population (Fig. 6F–H). Consistently, MLN4924 treatment also significantly decreased the absolute number of Granzyme B + and IFNγ + cells, while the combination with MEK inhibitor or PDL1 antibody significantly boosted the Granzyme B + and IFNγ + cells (Fig. S7D–G). Collectively, these results indicate that MLN4924 enhances cancer-associated immunosuppression through upregulation of PD-L1, thus compromising its anti-cancer activity, which is overcame by either MEK inhibitor or anti-PD-L1 antibody *via* PD-L1 targeting.

## Discussion

MLN4924 was first reported in 2009 as a potent small molecule inhibitor of neddylation activating enzyme, thus blocking the entire neddylation modification and inactivating the entire family of CRLs, which requires cullin neddylation for their enzymatic activities [6, 32]. Given that both neddylation modification and CRLs are over-activated in many types of human cancers, MLN4924 has shown in preclinical studies promising anti-cancer activities in a variety of human cancer cell lines [5], leading to its advancement to multiple Phase I-III clinical trials as a single agent or in combination with chemotherapies in the treatment of both hematological malignancies and solid tumors [33]. Notably, MLN4924 was approved by the FDA in 2020 as the breakthrough therapy based upon the survival benefits when administrated with azacitidine in patients with higher-risk myelodysplastic syndromes (MDS)/chronic myelomonocytic leukemia (CMML) or low-blast acute myeloid leukemia (AML) [34]. However, several studies have shown that MLN4924 has some off-target side-effects (for review see [14]), which could compromise its anti-cancer activity.

In this study, we found that MLN4924 increased PD-L1 levels in dose- and time-dependent manner, which is a general phenomenon, as seen in multiple human cancer lines (Fig. S1A–G). Our mechanistic study revealed that this could be yet another potential off-target effect of MLN4924 through activation of AP-1 *via* the ERK1/2 and JNK signaling pathways. Our conclusion is supported by the following lines of evidence: (1) MLN4924 caused a dose- and time-dependent induction of PD-L1 mRNA, suggesting a regulation at the transcriptional level; (2) MLN4924 activated ERK1/2 and JNK, while inducing PD-L1, and MLN4924 induction of PD-L1 can be abrogated by the inhibitors of MEK or JNK as well as by knockdown of *ERK1/2* or *c-JUN*, indicating a MEK/JNK dependent mechanism; (3) Abrogation of MLN4924 induction of PD-L1 by MEK inhibitor could not be blocked by proteasome inhibition, suggesting a degradation-independent mechanism; (4) PD-L1 transcriptional activation was dependent of AP-1 binding on the enhancer fragment. Thus, in addition to previously reported PD-L1 regulation in a manner dependent of neddylation and CRL1/3 [9, 23], PD-L1 induction by MLN4924 is also mediated by its off-target effect *via* activation of the MEK-JNK-AP1 axis. It is worth-noting that increase of PD-L1 protein levels is more than that of mRNA levels after MLN4924 treatment at the early stage (Fig. 1A–D), suggesting that neddylation-dependent (*via* blocking ubiquitylation and degradation) plays a major role, whereas neddylation-independent (*via* transcription activation by the MEK-JNK-AP1 axis) occurs later.

Although two recent studies have shown that MLN4924 treatment induces cancer-associated immunosuppression by upregulating PD-L1 expression in glioma cells [9, 22], our current study provided several lines of new finding: (1) PD-L1 induction can be achieved through activation of the MEK-JNK-AP1 axis by MLN4924, which is neddylation-independent; (2) MLN4924 induction of PD-L1 is a general phenomenon seen in multiple cancer cell lines derived from a variety of cancer types, likely through both neddylation-dependent (ubiquitylation and degradation) and independent (transcriptional activation) manners; (3) MLN4924 induction of PD-L1 has negative impact on its anticancer activity, which can be overcome by co-treatment with either MEK inhibitor or PD-L1 Ab.

PD-L1 is a major inhibitory checkpoint that negatively regulates the T-cell functions [15, 35, 36]. Cancer cells with high expression of PD-L1 evade T-cell anti-cancer immunity through the PD-L1/PD-1 blocking axis. As such, the therapeutic antibodies against PD-L1 or PD-1 have shown impressive anti-cancer efficacies in the treatment of various types of human cancers [37]. PD-L1 upregulation by cancer cells in response to drug exposure is likely to be a general phenomenon and is part of a pro-survival program. As such, delivery of chemotherapeutic treatment with immune checkpoint inhibitors may result in a more optimal outcome [38]. It has been shown that PD-L1 expression is subjected to regulation at the multiple levels, including transcription, post-transcription, and post-translational modifications (for review, see [39]). Notably, an AP-1 binding *cis*-element was identified in the enhancer region of *PD-L1* (+4785 to +5056 from transcription start site), and the MAPK signal appeared to upregulate PD-L1 expression depending on the AP-1 activity [28, 29]. Consistently, we found that MLN4924 induction of PD-L1 expression is in a manner dependent of this *cis*-element, further supporting the transcriptional regulation of PD-L1 expression in an AP-1 dependent manner. We also acknowledged that other transcription factors or oncogenic pathways may contribute to PD-L1 transcription, in addition to MYC, EGFR, PTEN/PI3K and MAPK [39, 40].

In the tumor microenvironment, PD-L1 is also expressed on various myeloid cell types [41], which contributes to immune escape by binding to PD-1 [42, 43]. Various anticancer therapies, including chemotherapy, radiotherapy, hormone- and targeted-therapies were able to modulate the recruitment of myeloid-derived suppressor cells, their activity and PD-L1 expression, resulting in immunosuppression [44]. Furthermore, cytotoxic chemotherapeutic agent cyclophosphamide can drive the expansion of myeloid cells, which exhibited the immunosuppressive function by tolerating CD4^+^ effector cells through the PD-1/PD-L1 axis [45]. Our previous study has shown that MLN4924 has immunosuppressive effect by suppressing the release of proinflammatory cytokines from dendritic cells, and mitigating dendritic cells-mediated T cell stimulation [46]. Here we showed that MLN4924 could compromise the immune function by inducing PD-L1 expression on cancer cells. It is reasonable to speculate that MLN4924 also induces PD-L1 expression on myeloid cells to cause immunosuppression. We, therefore, pursued this potential “side-effects” of MLN4924 biologically using both in vitro and in vivo tumor models. In an in vitro experiment with co-culture of T-cells and cancer cells, we observed that MLN4924 induction of PD-L1 in cancer cells triggered apoptosis of co-cultured Jurkat cells, which was abrogated by MEK inhibitor *via* blocking PD-L1 upregulation. On the other hand, activated T cells killed cancer cells, which was blocked by MLN4924 pretreatment. This MLN4924 blockage was reversed by either anti-PD-L1 antibody or MEK inhibitor. Thus, through PD-L1 induction, MLN4924 indeed negatively regulated anti-cancer activity of T cells, which can be abrogated by PD-L1 targeting.

We further tested this off-target “side-effect” of MLN4924 using an in vivo synergistic BALB/c mouse xenograft tumor model. Unlike in many immune-deficient nude-mice xenograft tumor models in which MLN4924 showed impressive anti-tumor activity by targeting neddylation and CRLs [47–49], MLN4924 had no effect on tumor growth in this immunocompetent syngeneic tumor model, indicating that its immune suppressive activity, as evidenced by induction of PD-L1 and reduction of toxic T cells in tumor tissues, counteracted its regular anti-cancer effect as a neddylation inhibitor [6, 32]. This notion is further supported by the observation that the combination of MLN4924 with either MEK inhibitor or PD-L1 (either one blocked immune-suppression) had enhanced anti-cancer activity, as compared to MLN4924 treatment alone. Taken together, MLN4924 conferred resistance of cancer cells to T cells-mediated cytotoxicity by inducing PD-L1 and the immune checkpoint, which is abrogated by blocking MEK pathway-regulated PD-L1 transcription or anti-PD-L1 antibody shown in both in vitro and one CT26 in vivo model. Human PD-L1 has 70% amino acid identity to its mouse orthologue. It was reported that human and mouse PD-L1 share enough sequence homology to allow for the interaction of the mPD-L1 with the hPD-1 protein, forming a functional immune checkpoint [50], which suggested that the results based on mouse models may have an application in humans. Thus, our study provided a sound rationale for effective anticancer therapy by the combination of MLN4924 with either MEK inhibitor or anti-PD-L1 antibody in future MLN4924-based clinical trials.

## Conclusions

**Fig. 7:**
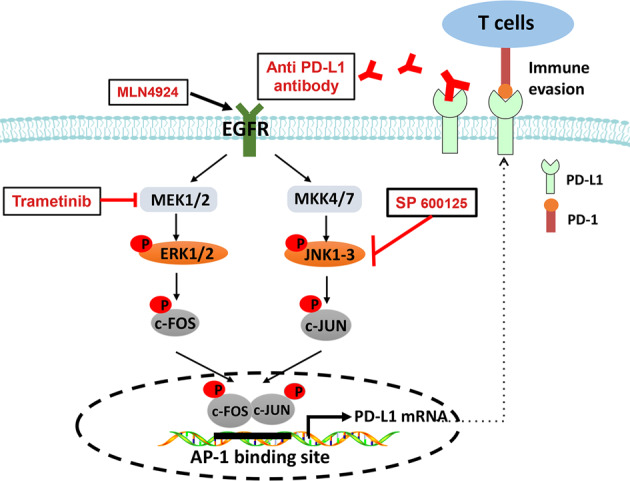
Working model. MLN4924 activates MEK1/2-ERK1/2 and MKK4/7-JNK kinases to induce c-FOS and c-JUN, respectively, leading to AP-1 activation. Activated AP-1 then binds to its *cis*-element in the PD-L1 enhancer sequence to transactivate PD-L1 expression. Accumulated PD-L1 contributes to cancer-associated immune evasion to counteract anti-cancer activity of MLN4924. This process can be inhibited by the inhibitor of MEK1/2 (trametinib), JNK (SP600125) and anti-PD-L1 antibody.

In summary, our study fits the following working model of MLN4924 induction of PD-L1 in a manner largely independent of neddylation-CRLs: MLN4924 activates MEK1/2-ERK1/2 and MKK4/7-JNK kinases *via* triggering EGFR dimerization [25] to induce c-FOS and c-JUN, respectively, leading to AP-1 activation. Activated AP-1 then binds to its *cis*-element in the *PD-L1* enhancer sequence to transactivate PD-L1 expression. Accumulated PD-L1 then induces cancer-association immune evasion to counteract anti-cancer activity of MLN4924. This process can be blocked by the inhibition of MEK1/2 (trametinib), JNK (SP600125) or anti-PD-L1 antibody (Fig. 7), suggesting the importance of combinational therapy for MLN4924-based cancer treatment.

## Materials and methods

### Cell lines and cell culture

H520, H1703, H2170, H358, H1792, H460, H1650, Het-1A, Kyse70, Kyse520, BxPC3, MC38, CT26 and Jurkat cells were maintained in RPMI 1640 media with 10% FBS and 1% penicillin-streptomycin. A549, H1299, SK-MES-1, MIA-PaCa2, PANC1, Capan1, M-Panc96, ID8, ID8-luci, B16-F0, B16-F10, Moh1 and UMSCCs were maintained in Dulbecco’s Modified Eagle’s Media (DMEM) with 10% FBS and 1% penicillin-streptomycin. Capan1 was maintained in Iscove’s Modified Dulbecco’s Medium with 10% FBS and 1% penicillin-streptomycin.

### Antibodies and reagents

The human PD-L1 (13684) Rabbit mAb, c-JUN (60A8) Rabbit mAb (9165S), Phospho-JNK (Thr183/Tyr185) (81E11) Rabbit mAb (4668T), Phospho-c-JUN (Ser63) II Antibody (9261S), NAE1 Rabbit mAb (14321S), p-ERK1/2 Rabbit mAb (4376S), ERK1/2 Mouse mAb (9106S), p-AKT Rabbit mAb (4060S), AKT Rabbit mAb (4691S), β-CATENIN Rabbit mAb (8480S), Cleaved-PARP Rabbit mAb (9541S) Cleaved-Caspase3 Rabbit mAb (9661S) and Phospho-c-FOS (Ser32) (5348T) were purchased from Cell Signaling Technology. UBA3 (ab124728) Rabbit mAb was purchased from Abcam. Mouse PD-L1 antibody (MAB90781-100) was purchased from R&D Systems. APC-conjugated anti-human PD-L1 (329708), APC anti-mice CD3 (100235), FITC anti-h/m Gramzyme B (515403), PE anti-mIFN-γ (505808), FITC-anti-mCD4 (10509), PE anti-mCD8a (100707), Fixation Buffer (420801) and Permeabilization Wash Buffer (10×) (421002) were purchased from Biolegend. Collagenase (C0130-500MG), β-ACTIN Mouse mAb (A5441) was purchased from Sigma. MEK inhibitor (trametinib) and JNK inhibitor (SP600125) were purchased from MedChem Express. AKT inhibitor (MK2206) and MLN4924 (S7109) were purchased from Selleck.

### Cell surface PD-L1 measurement

For tumor cell surface PD-L1 measurement, cells were collected and suspended in 100 µL of cell staining buffer and then incubated with APC-conjugated anti-human anti-PD-L1 antibody at room temperature for half an hour. Cells were then washed and stained in the staining buffer before analyzed by FACS.

### Quantitative real-time PCR

Total RNA was isolated by using TRIzol reagent (15596026, Thermo Fisher Scientific). cDNA was generated by reverse transcription using the Maxima First Strand cDNA Synthesis Kit for RT-qPCR (Thermo Fisher Scientific) as previously described [51]. The primers used for qPCR are as follows: the human *PD-L1*-Forward: ATTTGGAGGATGTGCCAGAG, Reverse: CCAGCACACTGAGAATCAACA. The mouse *Pd-l1-*Forward: GCTCCAAAGGACTTGTACGTG, Reverse: TGATCTGAAGGGCAGCATTTC. Relative mRNA levels were normalized to GAPDH levels. The human *GAPDH*-Forward: AAGGTGAAGGTCGGAGTCAA, Reverse: AATGAAGGGGTCATTGATGG. The mouse *Gapdh*-Forward: CCTGGA GAAACCTGCCAAGTATG, Reverse: GGTCCTCAGTGTAGCCCAAGATG). The samples were run in triplicates.

### Transfection of siRNAs

Cells were transfected with siRNAs (synthesized by Dharmacom, Lafayette, CO), using Lipofectamine 2000 (Invitrogen) according to the manufacturer’s instructions. The sequences of siRNA targeting UBA3 or NAE1 and scrambled control siRNA (siCont) are as follows:

si-Cont: 5′-AUUGUAUGCGAUCGCAGAC-3′,

si-UBA3#1: 5′-GCUACCAGAACACUGTAUU-3′,

si-UBA3#2: 5′-GCUUCUCUGCAAAUGAAAU-3′,

si-NAE1#1: 5′-CCAGGAGTATCTAACTATCAA-3′,

si-NAE1#2: 5′-GCATGTCACAAACTTCAGCAA-3′.

The siRNAs of c-JUN (sc-29223) were purchased from Santa Cruz Biotechnology. The siRNAs of p44/42 MAPK (ERK1/2) (6560S) were purchased from Cell Signaling Technology.

### Western blotting

Cells were lysed in cell lysis buffer contained with protease inhibitor cocktail. Proteins were separated on 7.5–12.5% SDS-polyacrylamide gels. Immunoblot analysis was conducted as described previously [52].

### Cell viability assay

Cells were seeded at 5000 per well in 96-well culture plates. MLN4924 was added to complete growth medium at concentrations ranging from 0.001 to 10 μM. After 72 h, Cell viability was measured using Cell Counting Kit-8 (CCK-8, MedChem Express) according to the manufacturer’s instructions and inhibitory concentrations were calculated.

### Chromatin immunoprecipitation (ChIP)

The pGL3 luciferase constructs containing either the PD-L1 promoter alone (PDL1-P), or the promoter plus the candidate enhancer (PDL1-P + E) were kindly provided by Margaret A. Shipp from Dana-Farber Cancer Institute and Harvard Medical School [28]. The ChIP assay was conducted by using the SimpleChIP^®^ Plus Enzymatic Chromatin IP Kit (9005, CST). In brief, cells were treated with or without MLN4924 for 48 h and fixed by adding formaldehyde into the culture medium to a final concentration of 1%. Fixed cells were washed and sonicated in a Branson 250 sonicator to produce genomic DNA fragments (100–400 bp). Then samples were immunoprecipitated with primary antibody of c-JUN (9165, CST) and protein A beads overnight at 4 °C. The beads were washed/eluted and phenol/chloroform-extracted and ethanol-precipitated. Finally, DNA was resuspended in 40 μL of water and applied for qPCR analysis. The sequence for PD-L1-ChIP primers are as follows: forward primer, 5′-TCACATTTCAAGCAGGATG ACTAAA-3′; and reverse primer, 5′-TGACTCACAGCCACTCTTCCA-3′.

### Co-culture study and T cell killing assay

Sub-confluent H358 or BxPC3 cells were treated with MLN4924 (0.5 μM) for 24 h. After medial removal and PBS wash, suspension of Jurkat (T lymphocyte cell line) cells (2×10E6) was added and co-cultured for 24 h. H358/BxPC3 and Jurkat cells were then harvested, separately for IB analysis. In T cell killing assay, H358 or BxPC3 cells were pretreated with MLN4924 for 24 h before co-culturing with Jurkat cells, pre-activated by phytohemagglutinin (PHA)/PMA at 10:1 ratio (Jurkat cells vs. cancer cells) in 24-well plates for 24 h. The suspension of Jurkat cells was then collected for Western blotting, whereas attached H358 or BxPC3 cells were either for Western blotting or stained with 0.5% crystal violet and photographed.

### The in vivo xenograft tumor model and drug treatment regimen

Animal studies were conducted and processed according to the guidelines established by the Zhejiang University Committee on Use and Care of Animals. The sample size of the animal experiment was based on the preliminary experiments and similar well-designed experiments, and no statistical method was used. BALB/C mice (6–8-week-old female, Jackson Laboratory) were subcutaneously injected with 1 × 10^7^ CT26 mouse colon cancer cells. One week after the injection, tumor-bearing mice were randomly divided into six groups after equalizing the average tumor size: IgG control antibody treatment (Vehicle group), MLN4924 treatment group, MEK inhibitor treatment group; MLN4924 and MEK inhibitor treatment group; anti-PD-L1 antibody treatment group and MLN4924 plus anti-PD-L1 antibody treatment group. Mice were injected intraperitoneally with 15 mg/kg MLN4924 (dissolved by 5% DMSO + 30% PEG 300 + 5% Tween 80 + ddH_2_O) or vehicle once a day, 5 days a week for 3 weeks. MEK inhibitor (trametinib) was intragastrically administrated with 1 mg/kg daily for 21 days. The anti-mouse anti-PD-L1 antibody (75 μg, 10 F.9G2, Bio X Cell, USA) or control rat IgG2b (LTF-2, Bio X Cell) was injected intraperitoneally every 4 days for a total of 5 injections. Tumor volumes were measured three times a week and calculated using the formula: length × width^2^ × 0.5.

### Single-cell generation from mouse tumor tissues and flow cytometry analysis

Mice tumor tissues were minced and digested with 5 ml collagenase (2 mg/ml) in DMEM for 1 h at 37 °C. Cells were collected by centrifugation and filtered through a 70 μm strainer before being lysed in a red blood cell lysis buffer for 5 min. Cells were then filtered through a 40 μm strainer in PBS with 2% BSA and then fixed in Fixation Buffer (420801, Biolegend) in the dark for 20 min at room temperature. The fixed cells were suspended in Intracellular Staining Perm Wash Buffer (421002, Biolegend) and centrifuged twice to permeabilize the cells. The permeabilized cells were then co-stained with antibodies against CD3 (100236, Biolegend), CD4 (100510, Biolegend) and CD8 (100708, Biolegend) or co-stained with antibodies against CD3, granzyme B (515403, Biolegend) or IFN γ (505808, Biolegend). The corresponding isotype IgGs were used for controls. After incubating with corresponding antibodies for 30 min at room temperature, cells were washed and analyzed by flow cytometry.

### Statistical analysis

The statistical significance of the differences was determined using *t*-test and one-way analysis of variance (ANOVA) for pairwise and group-wise comparisons, respectively, in GraphPad PRISM version 6 (GraphPad). The results were expressed as the mean ± SEM from at least three independent experiments. The tumor growth rates were estimated and compared using linear mixed regression models, in which the nested random effects were used to account for the longtidunal measurements of two tumors within each mouse, analyses were performed using R (version 4.0.1) [53]. For all analyses, statistical significance was defined using two-sided *P* values as *p* < 0.05 (*), *p* < 0.01 (**).

## Supplementary information

Supplemental Figure Legends

aj-checklist

Figure S1

Figure S2

Figure S3

Figure S4

Figure S5

Figure S6

Figure S7

## Data Availability

The datasets used and analyzed during the current study are available from the corresponding author on reasonable request.
