# Supplementary material for: PD-L1 induction via the MEK-JNK-AP1 axis by a neddylation inhibitor promotes cancer-associated immunosuppression
Source: Cell Death Dis. 2022 Oct 3;13(10):844. doi: 10.1038/s41419-022-05292-9 (PMC9529958; doi:10.1038/s41419-022-05292-9)
Supplement: Supplementary file 1 — Supplemental Figure Legends [file 41419_2022_5292_MOESM1_ESM.docx]

**PD-L1 induction via the MEK-JNK-AP1 axis by a neddylation inhibitor promotes cancer-associated immunosuppression**

**Running title:** PD-L1 induction by the MEK-JNK-AP1 signal

Shizhen Zhang ^1,2^, Xiahong You ^3^, Tiantian Xu ^3^, Qian Chen ^3^, Hua Li ^6^, Longyu Dou ^3^, Yilun Sun ^6^, Xiufang Xiong ^1,3^, Morgan A. Meredith ^6^, and Yi Sun ^1,3,4,5*^

^1^ Cancer Institute, the Second Affiliated Hospital, Zhejiang University School of Medicine, Hangzhou 310029, China.

^2^ Department of Breast Surgery and Oncology, Key Laboratory of Cancer Prevention and Intervention, Ministry of Education, the Second Affiliated Hospital, Zhejiang University School of Medicine, Hangzhou 310029, China.

^3^ Institute of Translational Medicine, Zhejiang University School of Medicine, Hangzhou 310029, China.

^4^ Zhejiang University Cancer Center, Hangzhou 310029, China.

^5^ Research Center for Life Science and Human Health, Binjiang Institute of Zhejiang University, Hangzhou, Zhejiang, 310053, China.

^6^ Division of Radiation and Cancer Biology, Department of Radiation Oncology, University of Michigan, 4424B MS-1, 1301 Catherine Street, Ann Arbor, MI 48109, USA.

**Supplemental Figure legends:**

**Fig. S1:** **MLN4924 increases PD-L1 levels in multiple human and mouse cancer cell lines**

**A-E**. Human esophageal and head & neck cancer cells (Kyse70, Kyse520, UMSCCs); pancreatic cancer cells (Moh1, MIAPaCa2, Capan1, BxPC3, M-Panc96), lung cancer cells (H1703, H358, H1792, H460, A549, H1650, H1299) and esophageal squamous epithelial cell (Het-1A) were treated with MLN4924 (0.5μM) for 24h, followed by IB with the indicated Abs. **F.** Murine cancer cell lines (ID8, ID8-luci, B16-F0, B16-F10, MC38, CT26) were treated with MLN4924 (0.5μM) for 24h, followed by IB with the indicated Abs. **G**. SK-MES-1 Cells were treated with MLN4924 (1 μM) for the indicated time periods, followed by IB with the indicated Abs. se: short exposure; le: long exposure.

**Fig. S2:** **MLN4924 induction of PD-L1 is neddylation independent, but ERK1/2-JNK pathway dependent**

**A.** *UBA3* knockdown had a minor effect on MLN4924-induced PD-L1 induction. SK-MES-1 cells were transfected with indicated siRNAs for 24 h, cells were then treated with MLN4924 (0.5μM) for 24 h, followed by IB with the indicated Abs. **B.** Both *NAE1* and *UBA3* knockdown had a minor effect on PD-L1 levels or MLN4924 induction of PD-L1. BxPC3 cells were transfected with siRNAs targeting NAE1, UBA3 or control siCont for 24 h, then treated with MLN4924 (0.5μM) for 24 h, followed by IB with the indicated Abs. **C.** Proteasome inhibitor MG132 had minor effect on PD-L1 levels. BxPC3 cells were treated with either MLN4924 (0.5 μM) for 24 h or MG132 (10 μM) for 8 h, followed by IB with the indicated Abs. **D.** MLN4924 induced phosphorylation of ERK1/2, AKT, JNK and c-FOS in dose- and time-dependent manners. BxPC3 cells were treated with various concentrations of MLN4924 for the indicated time periods, followed by IB with the indicated Abs. **E-F.** MLN4924 induction of both PD-L1 protein (Top) and mRNA (bottom) can be largely abrogated by inhibitors of MEK/JNK signal pathways (**E**), or siRNAs targeting these pathways (**F**). BxPC3 cells were treated with either MLN4924 (0.5 μM) or indicated inhibitors or both (**E**), or transfected with siRNAs targeting ERK1/2, c-JUN, alone with siCont. (**F**), followed by MLN4924 (0.5 μM) treatment for 24 hr, and then subjected to IB with the indicated Abs. MEKi: MEK inhibitor (trametinib); JNKi: JNK inhibitor (SP600125); AKTi: AKT inhibitor (MK2206). **G**. MEK inhibitor-mediated abrogation of PD-L1 induction by MLN4924 occurs at the transcriptional level. BxPC3 cells were treated with MLN4924 alone or combined with indicated inhibitors, followed by IB with the indicated Abs. MG132: proteasome inhibitor; CQ: lysosome inhibitor.

**Fig. S3: MLN4924 increases PD-L1 expression through ERK1/2-JNK pathway independent of MLN4924 concentrations**

**A-B.** MLN4924 IC_50_ curves in H358 (**A**) and BxPC3 (**B**) cells were generated. Cells were seeded at 5000 per well in 96-well culture plates. MLN4924 was added to complete growth medium at concentrations ranging from 0.001 to 10 μM. After 72 h, cell viability was measured using Cell Counting Kit-8 and inhibitory concentrations were calculated. **C-D.** H358 (**C**) and BxPC3 (**D**) cells were treated by MLN4924 at the broad range of IC50 concentrations (IC_20/40/60/80_) in the absence or presence of MEK inhibitor (100nM), followed by IB with the indicated Abs. **E-F.** H358 (**E**) and BxPC3 (**F**) cells were treated by MLN4924 at the broad range of IC50 concentrations (IC_20/40/60/80_) in the absence or presence of JNK inhibitor (20μM), followed by IB with the indicated Abs. MEKi: MEK inhibitor (trametinib); JNKi: JNK inhibitor (SP600125).

**Fig. S4: AP-1 activity controls the MLN4924 induction of PD-L1**

**A-B**. The binding of c-JUN to the PD-L1 enhancer sequence, enhanced by MLN4924. BxPC3 cells were treated with MLN4924, along with DMSO control, followed by ChIP-coupled PCR assay. The results were represented as % input (**A**) and Fold Enrichment (**B**). **C.** MLN4924 induction of PD-L1 depends on AP-1 binding site. BxPC3 cells were transfected with the indicated plasmids and then treated with MLN4924 (0.5 μM) for 24 h, followed by luciferase-based transcriptional assay. **D-E.** H358 (**D**) and BxPC3 (**E**) cells were transfected with the PDL1-P+E plasmids for 24h, and then treated with MLN4924 (0.5 μM) for 48 h, followed by luciferase-based transcriptional assay. Shown are mean ± SEM of three independent experiments. *p* < 0.05 (*), *p* < 0.01 (**)

**Fig. S5. MLN4924 attenuates cytotoxic effect of Jurkat cells through PD-L1 induction**

**A.** Jurkat cells co-cultured with MLN4924-pretreated cancer cells underwent apoptosis. Sub-confluent BxPC3 cells were treated with MLN4924 (0.5 μM) for 24 h. After medial removal and PBS washing, suspension of Jurkat cells was added and culture for 24h. BxPC3 and Jurkat cells were then harvested, separately, for IB with the indicated Abs. **B.** MLN4924-pretreated cancer cells became resistant to the killing by activated Jurkat cells. BxPC3 cells were treated with MLN4924 (0.5 μM) for 24 h, co-cultured with PHA/PMA activated Jurkat cells with or without PD-L1 ab for 24 h, followed by crystal violet staining of viable adhered BxPC3 cells and photography. Shown are mean ± SEM of three independent experiments. *p* < 0.05 (*), *p* < 0.01 (**).

**Fig. S6. MEK inhibitor enhances cytotoxic effect of Jurkat cells on MLN4924-pretreated cancer cells**

**A.** BxPC3 cells were treated with either MLN4924 (0.5 μM) or indicated inhibitors or both for 24h, then co-cultured with Jurkat cells for 24 h. BxPC3 and Jurkat cells were separately harvested for IB with the indicated Abs. **B.** BxPC3 cells were treated with either MLN4924 (0.5 μM) or indicated inhibitors or both for 24h, co-cultured with PHA/PMA-activated Jurkat cells for 24 h, followed by crystal violet staining and photography. Shown are mean ± SEM of three independent experiments. *p* < 0.05 (*), *p* < 0.01 (**). NC: negative control without Jurkat co-culture nor drug treatment.

**Fig. S7. Enhanced tumor suppression by the combination of MLN4924 with MEK inhibitor or PD-L1 antibody in synergistic BALB/c xenograft tumor model**

**A**. CT26 cells were treated with either MLN4924 or indicated inhibitors or both, followed by IB with the indicated Abs. **B**. Images of representative tumors for all six groups (Control, MLN4924, MEKi, PD-L1 ab, MLN4924+MEKi, MLN4924+ PD-L1 ab) after resected from mice. **C**. Immunoblot analysis of 4 individual tumor lysates from six indicated groups with indicated Abs. **D-G**. FACS profiling of tumor infiltrated T-cell population: Single cell suspension of tumor mass was prepared from each treatment group, and subjected to FACS profiling with indicated Abs. The data were quantified and statistically analyzed. MEKi: MEK inhibitor (trametinib); *p* < 0.05 (*), *p* < 0.01 (**); NS, not significant.
