# Supplementary figures and images for: PD-L1 induction via the MEK-JNK-AP1 axis by a neddylation inhibitor promotes cancer-associated immunosuppression
Source: Cell Death Dis. 2022 Oct 3;13(10):844. doi: 10.1038/s41419-022-05292-9 (PMC9529958; doi:10.1038/s41419-022-05292-9)

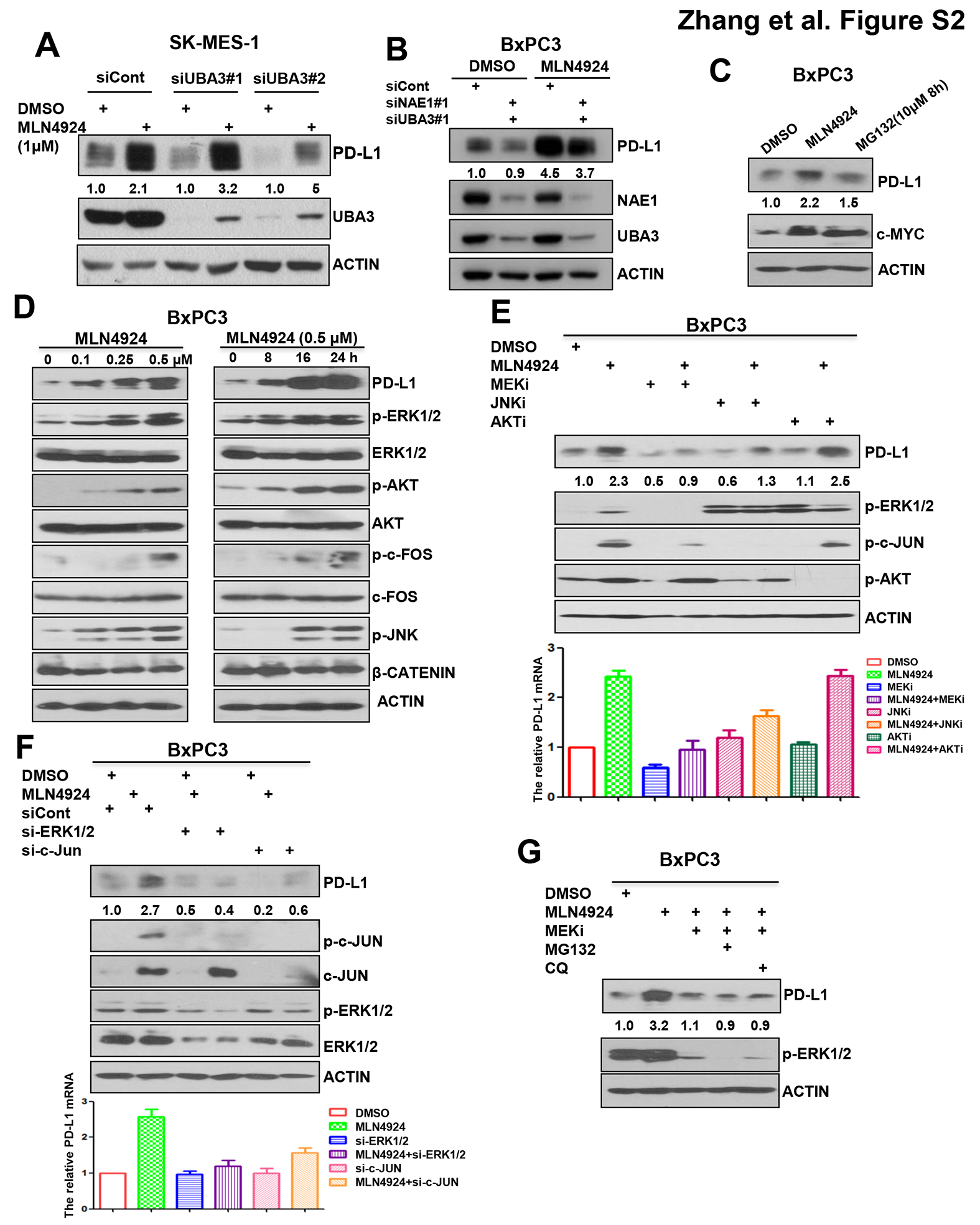

Supplement: Supplementary file 4 — Figure S2 [file 41419_2022_5292_MOESM4_ESM.tif]

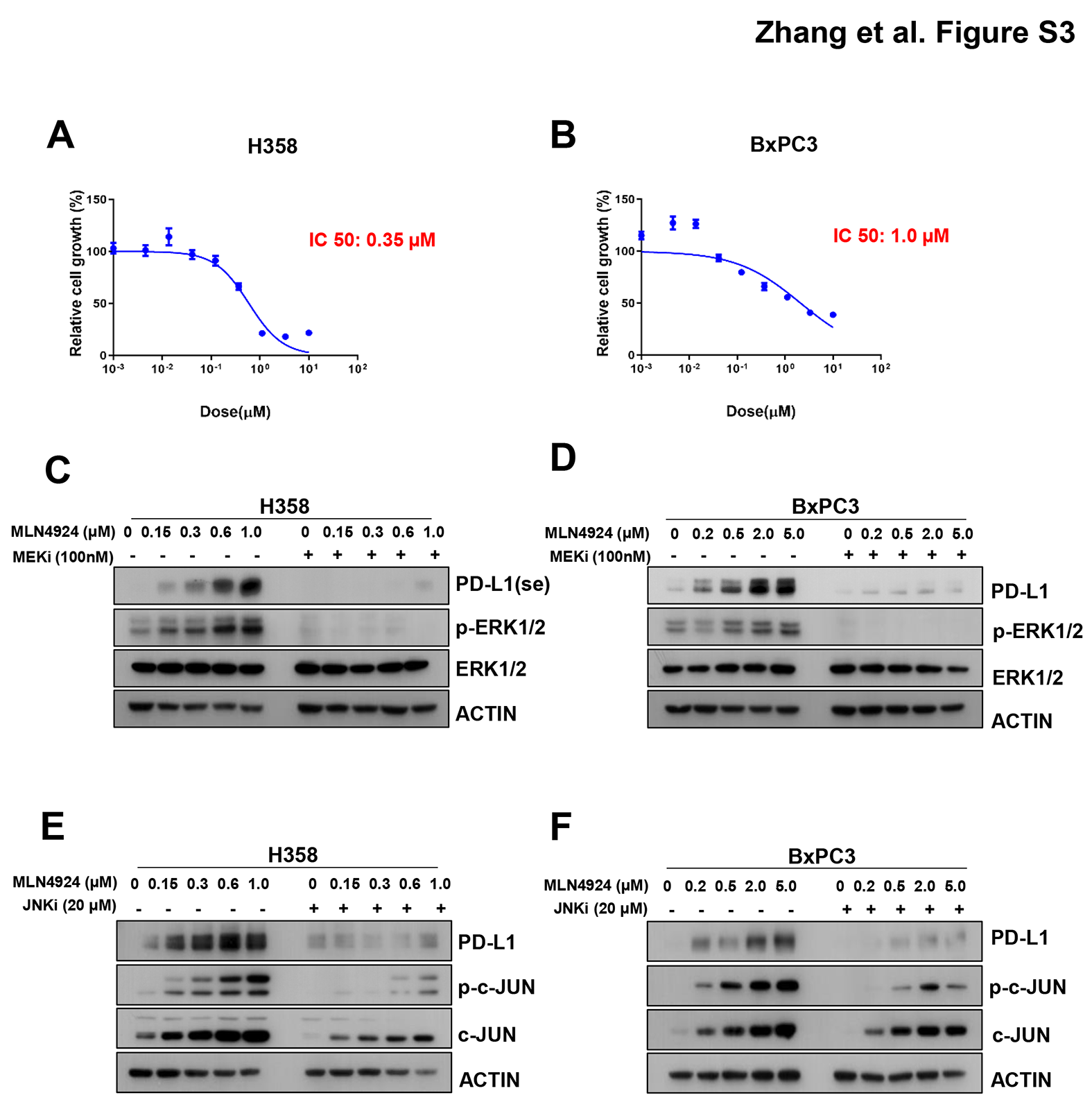

Supplement: Supplementary file 5 — Figure S3 [file 41419_2022_5292_MOESM5_ESM.tif]

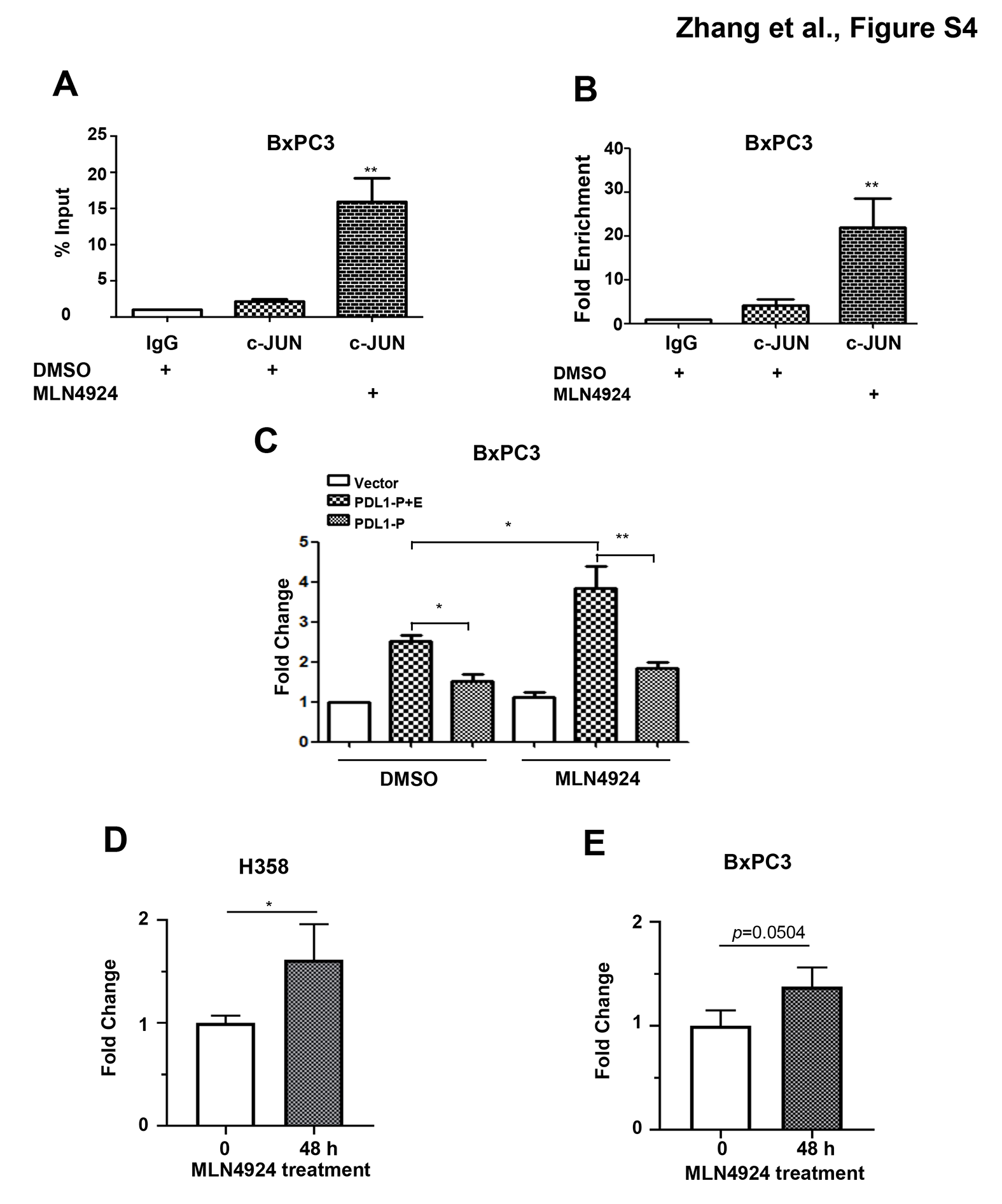

Supplement: Supplementary file 6 — Figure S4 [file 41419_2022_5292_MOESM6_ESM.tif]

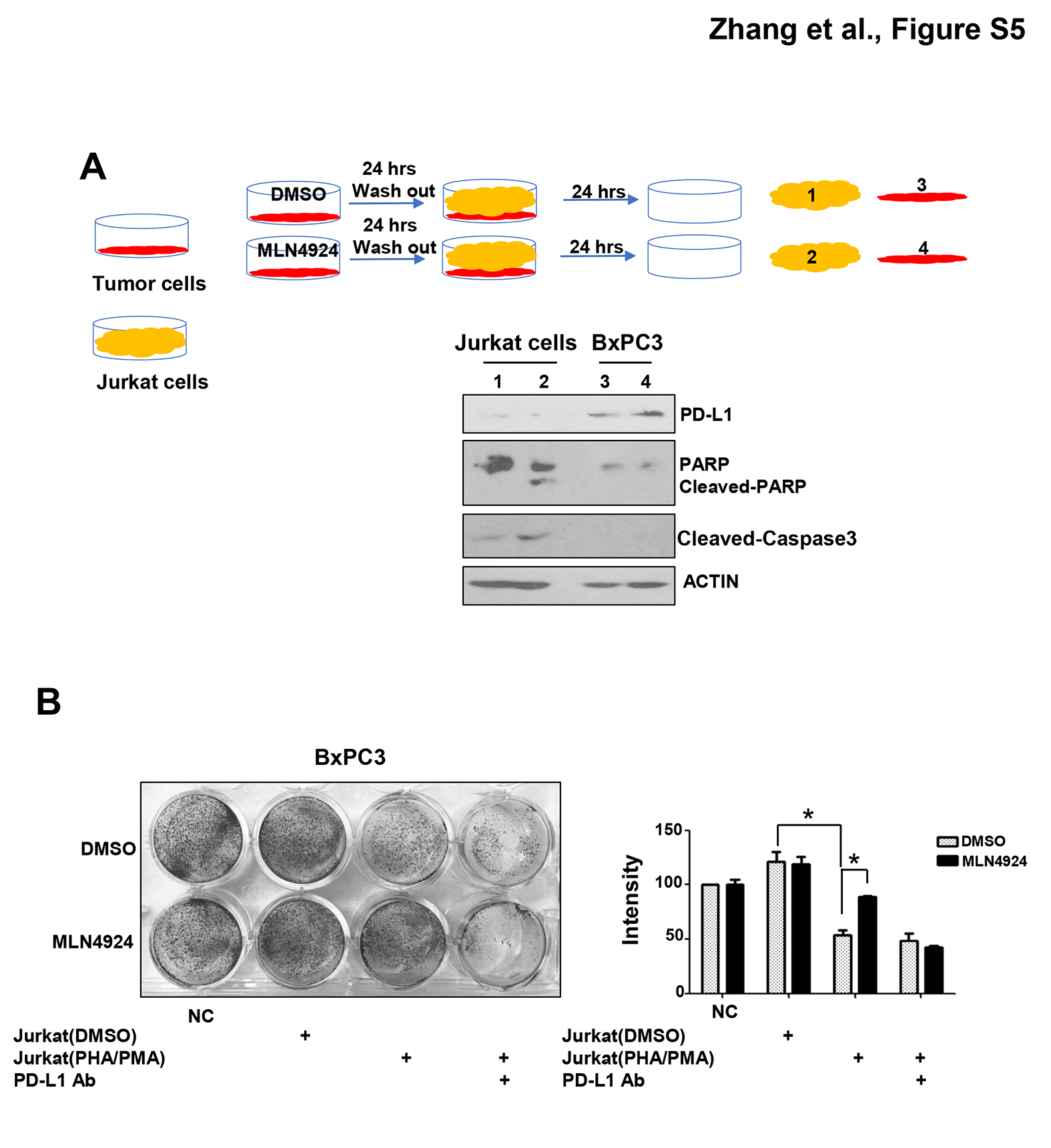

Supplement: Supplementary file 7 — Figure S5 [file 41419_2022_5292_MOESM7_ESM.tif]

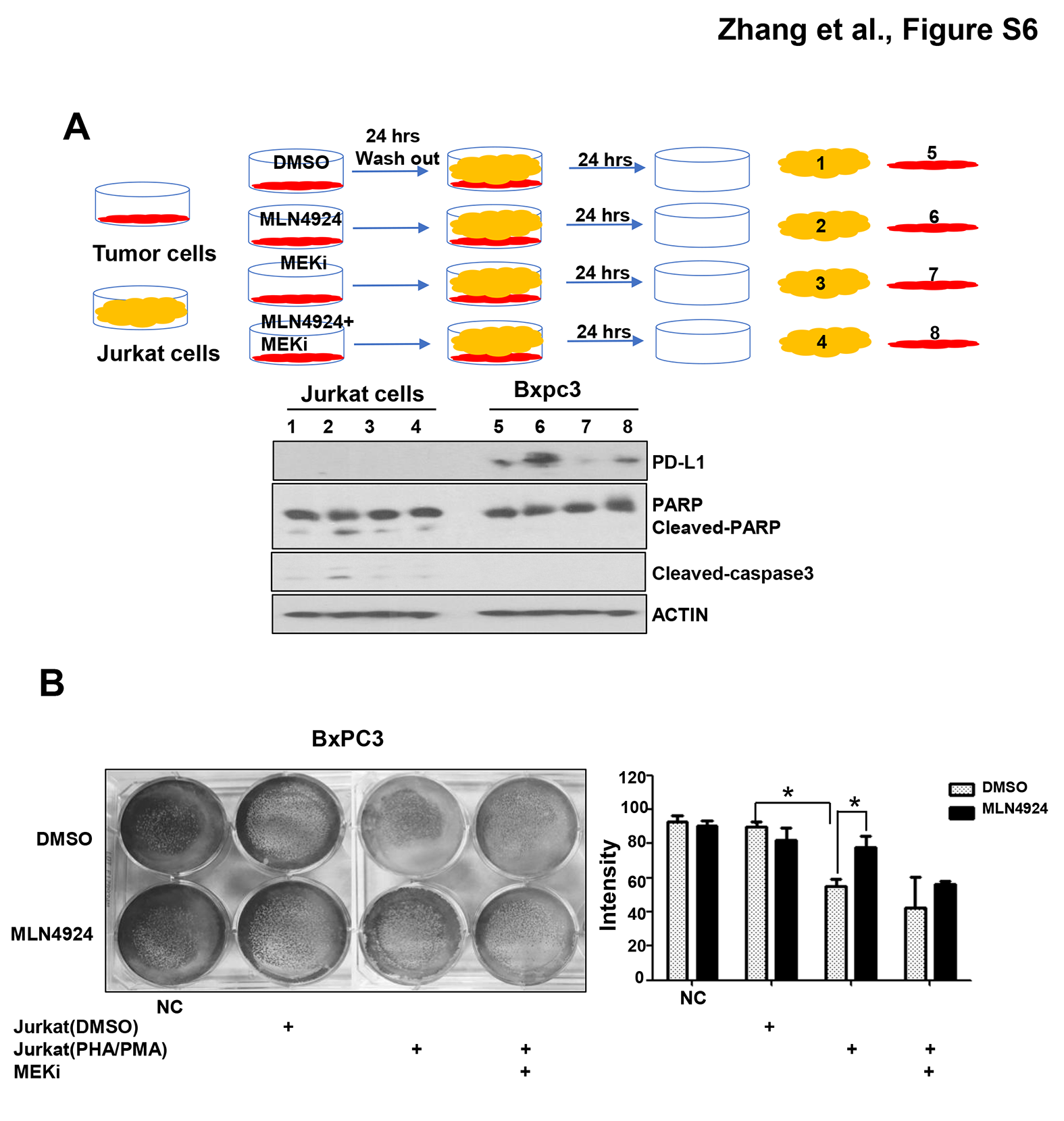

Supplement: Supplementary file 8 — Figure S6 [file 41419_2022_5292_MOESM8_ESM.tif]

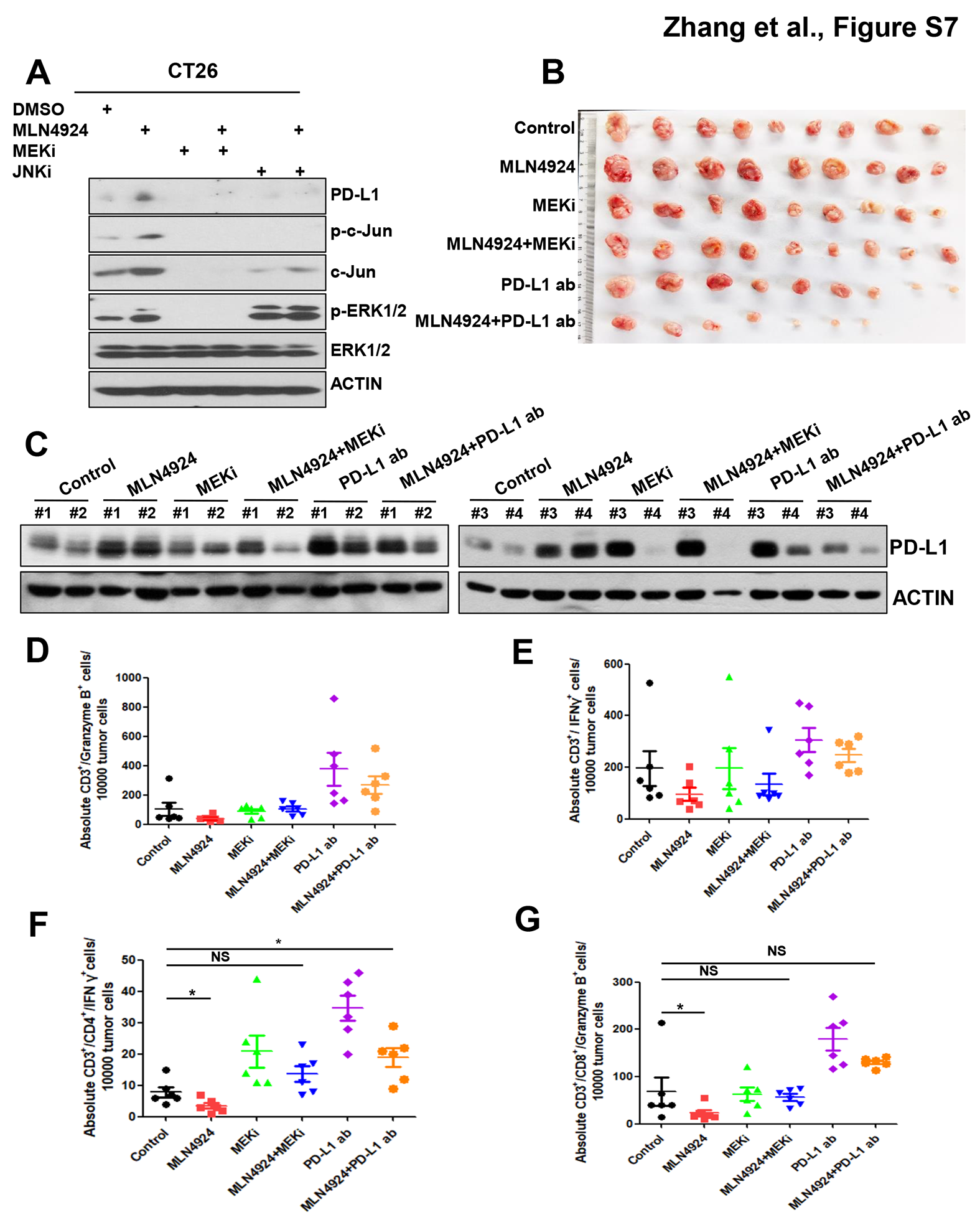

Supplement: Supplementary file 9 — Figure S7 [file 41419_2022_5292_MOESM9_ESM.tif]
